# Effects of Social Support and Self-Efficacy on Maternal Prenatal Cares Among the First-Time Pregnant Women, Iranshahr, Iran 

**Published:** 2017-06

**Authors:** Hossien Izadirad, Shamsoddin Niknami, Iraj Zareban, Alireza Hidarnia

**Affiliations:** 1 Department of Health Education and promotion, School of Medical Sciences, Tarbiat Modares University, Tehran, Iran; 2 Health Promotion Research Center, Zahedan University of Medical Sciences, Zahedan, Iran

**Keywords:** Pregnant Women, Self-Efficacy, Social Support

## Abstract

**Objective:** Social support and perceived self-efficacy affect health-related behaviors and play an important role on mothers' adaptability with pregnancy. This paper aims to study the impact of educational interventions based on social support and perceived self-efficacy on maternal prenatal care.

**Materials and methods:** The present study is a before after experimental study in which 90 first-time pregnant women were randomly selected and divided into two 45- participants experimental and control groups. Data were collected from 21 January to 20 May 2016. Determining the validity and reliability of the questionnaire, we used the panel of experts and Cronbach's alpha. The data collected from the two groups were compared before and 3 months after intervention and were analyzed by SPSS 18.

**Results:** Unlike the control subjects, there was a significant difference in maternal prenatal cares before and after an educational intervention between the scores of social support and perceived self-efficacy in the experimental group (p < 0.05). Before intervention, the average score of the experimental group was 12.62 ± 2.63 that rose to 17.71 ± 1.56, three months after the educational intervention, which is statistically significant (p < 0.05). There was a direct and positive relation between self-efficacy and maternal prenatal cares (p = 0.000, r = 0.538). Social support and self-efficacy predicted the variance of maternal cares by 69.2%.

**Conclusion:** Developing an educational program based on social support and perceived self-efficacy on maternal prenatal cares is helpful and efficient. The health system, family and society are in charge of making facilities and opportunities to improve social support and perceived self-efficacy in pregnant women, resulting in improved maternal prenatal cares.

## Introduction

Pregnancy is one of the natural events in any woman's childbearing age (1). Maternal prenatal cares can decrease the risk of death and complications from pregnancy and childbirth through recognizing and decreasing potential risks of pregnancy and contributing to women to correct the behavioral factors causing adverse pregnancy outcomes (2). Lack of adequate care of pregnant women during pregnancy can not only make problems for the pregnant women's health, but it also can result in problems for infants, including abortion, premature birth, low birth weight, and many other problems (3). Zhianian et al showed that the maternal prenatal cares for the pregnant women in Baluchestan is not sufficient (44%) (4). Different works have studied the impact of social support and self-efficacy and their relationship with maternal prenatal cares during pregnancy, type of delivery, and life quality (5, 6). The results have indicated that perceived social support, especially from husband, has positive effects on different aspects of self-care activities in pregnant women (7-9). Concerning the importance of social support and husband's supportive role that can reduce pregnancy complications (10,11) on one hand, and considering this fact that one of the barriers to maternal prenatal cares in the participants was the lack of support by husbands on the other hand, it is of great importance to study this issue. A pregnant woman not only needs social support, but also she should perceive her ability to pregnancy, maternal prenatal cares, and the new condition (6). Self-efficacy plays an important role in maternal transition into motherhood (12). It is based on the idea that one thinks s/he is able to organize events in order to reach self-desirable situation through proper manners and behaviors (13). When people with low level of self-efficacy face difficulties, they easily become convinced that their behavior is inefficient and stop trying their best. However, those with high self-efficacy pass difficulties with no trouble due to their skills and management; they aim to achieve during their lives resulting in better behavioral outcome compared to people with low level of self-efficacy (14). If a pregnant woman does not receive support well, not only will she experience low maternal parental self-efficacy, but the well-being of both the mother and her child may be affected. Hence, to improve the maternal and child health, it is significant to understand the influencing factors (15). As self-efficacy is a component to help understanding people’s behavior and to maintain heath promotional behaviors and due to social support roles in healthy behaviors during pregnancy (7). 

Considering the importance of the implementation of interventions such as educational interventions to improve maternal prenatal cares, this paper aims at studying the effects of educational interventions based on social support and self-efficacy on maternal prenatal cares in first-time pregnant women in Iranshahr, Iran.

## Materials and methods

***Population: ***This study is an intervention study. The methodology used in this paper is before after experimental method (controlled). The population consists of all firs-time pregnant women who referred to health care centers in Iranshahr (in Baluchestan Province) in 2016. The inclusion criteria were first-time pregnancy, wanted pregnancy, first marriage, monogamy, aged between 18-35, gestational age less than 13 weeks, a resident of the city of Iranshahr, having health care records and being healthy. The exclusion criteria were abortion, transferring to another center or town, and absence of the pregnant woman or her husband in training sessions. The size of samples, according to similar studies in Sistan and Baluchestan province (4), was evaluated as 45 subjects in each group (experimental and control) in 95% confidence level ${(Z}_{1-\frac{\alpha }{2}}$=1. 96), 80% statistical power (Z_1-B_ = 0.85), µ1=6.69, µ2 = 5.42, S1 = 1.86, S2 = 1. 39 and 20% probability of loss (according to social and cultural issues in Baluchestan). Sampling was multistage such that 2 (out of 5) urban health centers were randomly selected and then, they were divided into two experimental and control groups by simple random sampling. 45 pregnant women with inclusion criteria from each group were randomly selected. According to the office of maternal care, in both health centers under study, from 305 pregnant women (154 in health care of Experiment group and 151 in the health center of control group), 162 pregnant women were qualified (83 in health care of Experiment group and 79 in the health center of control group). In general, the rate of qualified people was 53.11%. Of 162 qualified women, 153 pregnant women (78 in health care of Experiment group and 75 in the health center of control group) have completed the written consent (participation rate 94.44%). In this study, the experimental and control groups were separately selected from health centers to prevent releasing information among them.

***Variables:*** The collecting data tool in this study was a self-made questionnaire including questions on demographic, self-efficacy, perceived social support and behavior. The questions on self-efficacy (7 questions), social support (8 questions), behavior (maternal prenatal cares) (8 questions) were evaluated by 3-option Likert scale. According to the instructions by the Ministry of Health, Treatment, and Medical Education, the maternal prenatal cares are: referring the pregnant woman to maternal and child health clinics (to measure weight, blood pressure, combined vaccination, etc.), physical activities, nutrition, ultrasound, prenatal testing, supplementation during pregnancy, oral health and participating in training classes. Evaluating the content validity of the questionnaire, we used the panel of experts and comments of 10 maternal and child health experts were exerted. Evaluating the reliability of the questionnaire, we used Cronbach's alpha coefficient (0.81). 

Selecting the experimental and control groups randomly, the self-made questionnaire was distributed among the subjects and the basic information was obtained. Then, an educational program, according to the pregnant women's cultural characteristics and literacy level, was implemented in order to improve the self-efficacy (increasing mothers' skills, using the successful models, such as the experiences of the other first-time pregnant women who could successfully do the maternal prenatal cares in the same society with similar conditions, educational videos, setting schedules, and verbal persuasion) in experimental subjects for two 40-50 minute sessions with a training manual. For the second target group, who were the pregnant women's husbands, a 60-minute training session was held in Health Center auditorium. Improving social support (emotional, informational, and financial supports as well as companionship with the pregnant woman, especially in the studied area where, according to need-assessment, lack of security is regarded as one of the reasons for failing in maternal prenatal cares), we held a lecturing training session and played an educational video for the husbands to emphasize on the importance of companionship, emotional, informational, and financial supports to have healthy pregnancy, mother and baby. Immediately after the intervention and 3 months after the intervention, the subjects answered the same questionnaire, through which the changes in perceived self-efficacy, social support, and behavior (maternal prenatal cares) were determined by statistical test. Finally, the data collected from the two groups before, immediately after, and 3 months after the educational intervention were encoded and analyzed. Considering the ethical principles, we obtained the subjects' and their husbands' informed consents and provided the referral letter to the health care center management. This study was conducted as a single blind, in this case that the analyst of statistics was not aware of the allocation of the groups to the Experiment and control groups. 

***Statistical Analysis: ***The data were analyzed by Spss software version 18. The repeated measurement test was used to compare mean scores of self-efficacy, social support and behavior, in three stages (before, immediately and three months after intervention), independent t-test and chi square test were used to examine the background variables between the two independent groups. Internal correlation of self-efficacy, social support and behavior was analyzed using Pearson correlation coefficient. The multivariate regression was used to determine the variables predictive of behavior,. The results were considered significant at a significant level less than 0.05.

***Ethical Approval:*** The study protocol received ethical approval from the Tarbiat Modares University in Tehran for Population Science’s Ethical (No: 52d/2245). Written informed consent was obtained from all respondents before starting the intervention.

## Results

The average age of the participants in experimental and control group was 20.78 ± 4.18 and 21 ± 2.97, respectively. According to the results of independent T test, there was no significant statistical difference in the average scores of social support, self-efficacy, behavior, and the average age between the experimental and control groups before the intervention. 

There was no significant statistical difference, according to chi-square test, in experimental and control subjects' education level, income, occupation, and insurance (Table 1). 

**Table 1:** Absolute and relative frequency distribution of demographic specifications in experimental and control pregnant women

**Variable**	**Category**	**Experiment**		**Control**		**p value**
		**Number**	**%**	**Number**	**%**	
Educational Level	Guidance school	14	31.11	17	37.77	0.266
	High school	16	35.55	20	44.44	
	Collegiate	15	33.33	8	17.77	
Womans occupation	Housewife	41	91.12	42	93.33	0.500
	Employee	4	8.88	3	6.66	
Household monthly income	Very weak ( less than 5 million Rial)	26	57.77	30	66.66	0.670
	Weak (between 5 million to 10 million Rial)	12	26.66	10	22.22	
	Average (between 10 to 20 million Rial)	7	15.55	5	11.11	
Insurance status	Have insurance	41	91.12	40	88.88	0.725
	Does not have	4	8/88	5	11.12	
Age(Mean ± SD)		20.78 ± 4.18		21 ± 2.97		0.722

**Table 2:** The comparison between the average, standard deviation, and significance level of social support, self-efficacy, and behavior before, immediately after, and 3 months after the intervention in experimental and control groups

**Variable**	**Group**	**Before ** **intervention**	**Immediately after ** **intervention**	**3 months after ** **intervention**	**p value** **Repeated Measuresment**
Self-efficacy	Experiment	9.756 ± 0.236	12.867 ± 0.243	13.156 ± 0.149	0.000
	Control	9.156 ± 0.248	9.222 ± 0.244	9.333 ± 0.234	0.201
Social support	Experiment	10.711 ± 0.271	15.044 ± 0.271	15.133 ± 0.263	0.000
	Control	10.622 ± 0.202	10.733 ± 0.221	10.756 ± 0.218	0.057
Behavior	Experiment	12.622 ± 0.392	14.044 ± 0.313	17.711 ± 0.233	0.000
	Control	12.60 ± 0.232	12.578 ± 0.272	12.533 ± 0.233	0.596

According to the results in Table 2, the scores of pre-test on social support, self-efficacy, and behavior were more moderate levels. However, after intervention, there was a significant and increasing difference in the experimental subjects while the increase in the experimental control subjects (who were trained) was inconsiderate and insignificant (Table 2). 

The correlation between social support, self-efficacy, and maternal prenatal cares was studied in this paper. The results are shown in Table 3.

Studying the predictive power of social support and self-efficacy, we used the multivariate regression analysis. Generally, the variables predicted 69.2% of maternal prenatal care behaviors, after the intervention. The variables of self-efficacy had the most predictive power (Table 4).

## Discussion

The results showed that educational intervention based on social support and self-efficacy are effective on increasing maternal prenatal care behaviors. Self-efficacy is a linking principle between knowledge, behavior, and believing one's abilities to do a behavior. Having knowledge about the reasons for doing a behavior is not sufficient, but one should feel abilities to do it. Perceived self-efficacy is the first step to do an activity and hence, it is of great importance (16). In this study, the average score of self-efficacy before the intervention was 9.75 (out of total 14 scores) in the experimental group. It rose to 12.86 immediately after the intervention and after 3 months of the intervention, it was 13.15, indicating a significant difference. This result conforms to the results of a study by Daryani, in which self-efficacy revealed a significant increase after the intervention (17). In the study by Song, it was revealed that the program of improving self-efficacy (including components of observational learning, behavior, and verbal persuasion) can have great impacts on self-efficacy (18). 

The findings suggest that the perceived social support, after the intervention in pregnant women in the experimental group was significantly more than the control group. In the study Mortazavi, Reports pregnant women in the experimental group, particular, more attention to their nutrition and their spouses to provide the necessary background for grocery shopping, confirms the results of our study (19).

The results of the present study showed that unlike the control subjects, the average of maternal prenatal care behaviors in the experimental subjects increased significantly (p < 0.001).

**Table 3:** Correlational Coefficient matrix of social support, self-efficacy, and behavior

	**1**	**2**	**3**	**4**	**5**	**6**	**7**	**8**	**9**
Self-efficacy before intervention	-								
social support before intervention	0.330*	-							
behavior before intervention	0.474**	0.195	-						
Self-efficacy immediately after intervention	0.550**	0.056	0.189	-					
Social support immediately after intervention	0.248	0.547**	0.065	0.339*	-				
behavior immediately after intervention	0.154	-0.086	0.205	0.055	-0.179	-			
Self-efficacy 3months after intervention	0.499**	0.050	0.213	0.919**	0.408**	-0.068	-		
social support 3months after intervention	0.207	0.593**	0.075	0.243	0.981**	-0.192	0.336*	-	
behavior 3months after intervention	0.348**	0.002	0.249	0.538**	0.180	0.233	0.583**	0.138	-

* p ˂ 0.05;

** p ˂ 0.01

**Table 4:** Regression Analyses

**Variable**	**B**	**SE**	**β**	**t**	**Sig.**	**R** ^2^	**p**
Constant	2.712	0.903		3.003	0.003	0.692	0.000
Self-efficacy	0.804	0.125	0.634	6.444	0.000		
Social support	0.264	0.109	0.237	2.413	0.018		

The results are in line with the results found by Khoramabadi who concluded theory-based educational intervention can improve the nutritional behavior in pregnant women (20). 

The results indicated a significant statistical relationship between self-efficacy and maternal prenatal care behaviors. Rise reported a significant statistical relationship between self-efficacy and health behavior (21). Also, the study by Zhianian revealed a significant relationship between self-efficacy and maternal prenatal care behaviors (4). There could be found no significant relationship between perceived social support and maternal prenatal care behaviors. Abdollah pour (22) found no significant relationship between social support and maternal prenatal care behaviors. Unlike the results of the present study, Teitler reported a significant statistical relationship between social support and maternal prenatal care (23). The differences in the results of the study by Teitler are caused by difference in the target group (formal marriage, romantic relationship, divorced, and cohabitant) as well as difference in types of social support (financial support by husbands). According to the results of this study, there is a significant relationship between social support and self-efficacy, being conformed by the results of the previous studies, which showed social support improves self-efficacy (24, 25). 

Moreover, the results of the present study show the predictive power of social support and self-efficacy on maternal prenatal care behaviors. Self- efficacy was more powerful, such that it can predict 63% of changes of maternal prenatal care behaviors. It seems that it is due to its direct influence on the target group (pregnant women) and recommendations by culture (especially by religion) on the responsibility of individuals, particularly in pregnancy. This issue is of great importance in many cultures such that one of the reasons of improving self-efficacy among Chinese women, especially on maternal prenatal cares, is recommendations by culture on people's accountability (26). 

Though social support had weaker predictive power than self-efficacy, it should be considered in interventions for improving maternal prenatal care behaviors since others' social support, especially husband's support, and the qualities of maternal prenatal cares can determine the quality of pregnancy, type of delivery, and fetal health (27). It seems that perceived social support as an influential factor that can have indirect impacts on maternal prenatal cares should be of great consideration in order to increase the impact of the interventions on maternal prenatal cares. In this regard, a study showed increased maternal prenatal cares caused by increased financial support by the husband (23). Elsenbruch showed that lack of social support is a risk factor for the mother's health in pregnancy and has harmful effects on pregnancy (27), and also, social support increase self-efficacy (24, 25). 

The results of the present study showed that the health program designed to improve perceived self-efficacy a social support as well as maternal prenatal care behaviors had great influence. In this context, the health system family, and society are in charge to provide facilities and opportunities to increase social support and perceived self-efficacy in pregnant women and contribute to improve maternal prenatal cares and the complications of pregnancy. 

Limitations of this study including the small sample size that may have provided inadequate statistical power to detect moderate by meaningful differences as statistically significant, the lack of representativeness of the sample that may have reduced the generalizability of findings, the lack of use of standard instruments that may have resulted in misclassification of information and/or lack of comparability of findings to those from other studies that have used standard instruments.

The strengths of this study are discussion, using pregnant women's experiences, and attracting the husbands' support, leading to a more friendly social relationship between pregnant women and their husbands, and supporting each other. The other important strength is emphasizing on improving self-efficacy and getting involved the people in self-care behaviors. The maternal prenatal cares by the pregnant woman makes her responsible for maintaining her health and increases her self-efficacy, while in other routine interventions, the role of self-efficacy, family, and especially the husband's role have been ignored.

***Conclusion:*** According to the results of the present study, if this project is implemented in all prenatal care clinics and health centers as a continuous intervention, it can be influential on improving the maternal prenatal cares, pregnant women's perceived social support, and self-efficacy. In this context, it is suggested to implement this project in order to evaluate long-term prenatal cares (by the end of pregnancy), complications of pregnancy, and delivery in a larger scale, and study its applicability in different areas.
